# Wrinkles Worries: Decoding Allergy Reactions to Botulinum Neurotoxin

**DOI:** 10.1111/jocd.70116

**Published:** 2025-03-17

**Authors:** Hurson Charlotte

**Affiliations:** ^1^ Hôpitaux Universitaire de Strasbourg Unité d'Allergologie Strasbourg France

**Keywords:** allergy, botulinum neurotoxin, delayed hypersensitivity, immediate hypersensitivity

To the Editor,

1

Botulinum neurotoxins (BontA) are commonly used in the treatment of various muscle hyperactivity syndromes, as well as for aesthetic purposes. The potential of these neurotoxins was highlighted in the early 1990s by Drs. Carruthers, a Canadian medical couple, who demonstrated the efficacy of OnabotulinumtoxinA (ONA) in the treatment of forehead wrinkles. BontA, secreted by the bacterium *Clostridium botulinum* , acts by inhibiting the release of acetylcholine at the neuromuscular junction, thereby preventing muscle activity and reducing the appearance of facial wrinkles. In France, three brands of botulinum toxin are currently approved for aesthetic use: AbobotulinumtoxinA (ABO), ONA, and IncobotulinumtoxinA (INCO). Despite their widespread use, BontA can lead to immunological complications, such as the formation of blocking antibodies, responsible for loss of efficacy [1] or immediate (type I) or delayed (type IV) hypersensitivity reactions (HS).

The aim of this paper is to review our current knowledge of allergic HS to BontA.

A comprehensive literature review was conducted on PubMed to identify all reported cases of immediate and delayed HS to botulinum toxin using the keywords “botulinum toxin”, “botulinum toxin type A”, “adverse effects”, “adverse drug reaction”, “delayed hypersensitivity”, “immediate hypersensitivity”, “anaphylaxis”, “allergy”, “case report”. The articles selected are those that provide a clinical or methodological description of paraclinical testing or an epidemiological analysis of BontA allergy.

Epidemiological data are limited. In 2000, the FDA (Food and Drug Administration) reported 1437 adverse events associated with BontA, of which 1031 were related to cosmetic use [2]. A systematic literature review carried out in 2018, based on an analysis of seven articles, revealed that the incidence of anaphylaxis after BontA injection was < 2%. No cases of fatal anaphylaxis were reported [3].

In 2020, the FDA Adverse Event Reporting System conducted between 2014 and 2019 reported allergy as the 5th most common adverse event (3.4%), after pain, edema, palpebral ptosis, and headache [4]. However, the diagnosis of allergy was not verified in all patients, and confusion between allergy and adverse or iatrogenic effects may have led to an overestimation of these figures.

Type I HS can lead to mucocutaneous reactions or outright anaphylaxis, while type IV HS causes toxidermia of varying severity. Treatment modalities are no different from those for other drug allergies, that is, anti‐histamines, bronchodilators, systemic corticosteroids, or intra‐muscular adrenaline for type I HS, or dermocorticoids and emollients for type IV HS. A rapid assessment of severity is crucial to ensure that patients are referred to an emergency department or hospital when necessary.

The first case of immediate HS to ONA was reported in 2005, but the imputability of BontA was not formally established due to the presence of lidocaine in the formulation [5]. Subsequently, other cases of HS type I have been reported, sometimes attributed to counterfeit products where the allergen could not be identified [6], or to excipients present in the injected products, as suggested in a patient who developed urticaria after BontA injection [7]. In this patient, the product used was produced in China and contained bovine gelatin, a potential allergen. The intradermal reaction (IDR) performed with the product in question was positive.

In some cases, diagnosis is made through a process of elimination, as demonstrated in the study by Moon, which examined a case of grade II anaphylaxis occurring 5 min after BontA injection and requiring intramuscular adrenaline administration [8]. The authors did not perform IDR with the neurotoxin itself but conducted IDR with saline solution and patch tests with the anesthetic cream used. Since these tests were negative, the neurotoxin was considered the likely cause.

A recent study analyzed the individual case safety reports (ICSRs) sent to the EudraVigilance database regarding immediate HS to BontA [9]. A total of 86 ICSRs reporting BontA as a suspected drug were retained: the majority of patients with BontA‐induced anaphylaxis were women (89.5%) and mostly in the 18–64 age group. The most frequent outcome was “recovered/resolved” while the most frequent severity criterion was “caused/prolonged hospitalization”. The authors of this study, therefore, suggest close monitoring before, during, and after intramuscular injection of BontA in order to ensure appropriate management of patients receiving this drug.

In 2014, Rosenfield’s team explored a rash that occurred 36 h after the 4th injection of BontA: prick tests and IDR were negative, but patch tests confirmed type IV hypersensitivity [10]. Unfortunately, the exact procedures for performing skin tests were not detailed.

More recently, Giraldo et al. investigated the case of a patient who developed pruritic erythematous plaques at the injection sites 48 h after BontA injection [11]. Skin tests with ONA, ABO, and INCO diluted to the 10th were performed and were negative.

In contrast, a lymphocyte transformation test (LTT) was performed and was positive for ONA and ABO but negative for INCO. Following this, the patient was treated with INCO without an adverse reaction.

Classically, immediate drug‐induced HS is investigated in vivo by prick‐tests followed by IDR, whereas delayed HS is investigated by IDR and patch‐tests: positive predictive values (PPV) and negative predictive values (NPV) depend on the drug studied.

In the 2018 systematic review, four studies including 302 patients who had undergone skin testing were analyzed: in three studies, the method practiced was “intradermal”; one study did not indicate the method use. Skin testing showed variable predictive value, ranging from 0% to 56% for PPV and from 50% to 100% for NPV [3].

In an American database, skin tests performed in 33 patients showed an NPV and PPV of 100%, but the method used is not detailed [3].

As far as in vitro tests are concerned, to date there are no specific IgE antibodies to BontA, and no basophil activation tests have been reported to explore type I HS. Only 1 case of type IV HS has been investigated using LTT [11].

To date, the literature on HS to BontA is limited and not very detailed. Both types of hypersensitivity, immediate and delayed, are rare, and skin testing procedures are not standardized. It is essential to consider the imputability of BontA itself, particularly after ruling out hypersensitivity to excipients, local anesthetics, or latex, if these have been used. In France, authorized products contain human albumin as a common excipient, potentially allergenic but not testable. ABO specialties also contain lactose, but no case of allergy to this product via this excipient has been reported. No risk factors for allergy to BontA have been identified, and there is no recommendation for predictive allergological testing.

Although allergic reactions to BontA are rare, they can be severe, highlighting the need for appropriate treatment options to be readily available in medical facilities that administer these injections. If an allergy is suspected, referral to an allergy department is recommended for in vivo and/or in vitro testing to identify the responsible molecule and explore alternative treatment options. Further research is essential to establish standardized protocols for conducting comprehensive allergological assessments.

## Conflicts of Interest

The author declares no conflicts of interest.

## Data Availability

The author has nothing to report.
